# Supplementary material for: Meta-Analysis and Gene Set Enrichment Relative to ER Status Reveal Elevated Activity of MYC and E2F in the “Basal” Breast Cancer Subgroup
Source: PLoS One. 2009 Mar 9;4(3):e4710. doi: 10.1371/journal.pone.0004710 (PMC2650420; doi:10.1371/journal.pone.0004710)
Supplement: Dataset S1 — GSEA results from first screen. The file contains a zipped archive of the HTML results of the second GSEA screen. Reports for enriched or depleted datasets may be accessed from the “index.html” link within the my_analysis.GseaPreranked.1219975950625.rpt.zip ZIP archive. Enrichment plots are available for each of the 50 most enriched and depleted gene sets. (13.32 MB ZIP) [file pone.0004710.s001.zip › my_analysis.GseaPreranked.1219975950625.rpt.zip!//index.html]

Index for xtools.gsea.GseaPreranked my\_analysis.GseaPreranked.1219975950625

### GSEA Report for Dataset zSco.ERneg.to.ERpos

#### Enrichment in phenotype: **na**

- 1813 / 2321 gene sets are upregulated in phenotype **na\_pos**- 529 gene sets are significant at FDR < 25%- 274 gene sets are significantly enriched at nominal pvalue < 1%- 454 gene sets are significantly enriched at nominal pvalue < 5%- Snapshot of enrichment results- Detailed enrichment results in html format- Detailed enrichment results in excel format (tab delimited text)- Guide to interpret results

#### Enrichment in phenotype: **na**

- 508 / 2321 gene sets are upregulated in phenotype **na\_neg**- 66 gene sets are significantly enriched at FDR < 25%- 46 gene sets are significantly enriched at nominal pvalue < 1%- 82 gene sets are significantly enriched at nominal pvalue < 5%- Snapshot of enrichment results- Detailed enrichment results in html format- Detailed enrichment results in excel format (tab delimited text)- Guide to interpret results

#### Dataset details

- The dataset has 22283 native features- After collapsing features into gene symbols, there are: 13321 genes

#### Gene set details

- Gene set size filters (min=15, max=1500) resulted in filtering out 589 / 2910 gene sets- The remaining 2321 gene sets were used in the analysis- List of gene sets used and their sizes (restricted to features in the specified dataset)

#### Gene markers for the **na\_pos** *versus* **na\_neg** comparison

- The dataset has 13321 features (genes)- Detailed rank ordered gene list for all features in the dataset

#### Global statistics and plots

- Plot of p-values *vs.* NES- Global ES histogram

#### Other

- Parameters used for this analysis- Zipped file with all results

---

Report: my\_analysis.GseaPreranked.1219975950625.rpt   by user: marall

xtools.gsea.GseaPreranked [Fri, Aug 29, '08 0 PM 12]

Website: www.broad.mit.edu/GSEA
Questions & Suggestions: Email
